# LAVAA: a lightweight association viewer across ailments

**DOI:** 10.1093/bioadv/vbad018

**Published:** 2023-02-15

**Authors:** Eric B Fauman, Stella Keppo, Nicola Cerioli, Rupesh Vyas, Mitja Kurki, Mark Daly, Mary Pat Reeve

**Affiliations:** Internal Medicine Research Unit, Pfizer Worldwide Research, Development and Medical, Cambridge, MA 02139, USA; Visual Communication Design, Aalto University, Espoo 02150, Finland; Visual Communication Design, Aalto University, Espoo 02150, Finland; Visual Communication Design, Aalto University, Espoo 02150, Finland; Finnish Institute for Molecular Medicine (FIMM), University of Helsinki, Helsinki 00014, Finland; Program for Medical and Population Genetics, Broad Institute of MIT and Harvard, Cambridge, MA 02142, USA; Stanley Center for Psychiatric Research, Broad Institute of MIT and Harvard, Cambridge, MA 02142, USA; Analytic and Translational Genetics Unit, Massachusetts General Hospital, Boston, MA 02114, USA; Center for Genomic Medicine, Massachusetts General Hospital, Boston, MA 02114, USA; Finnish Institute for Molecular Medicine (FIMM), University of Helsinki, Helsinki 00014, Finland; Program for Medical and Population Genetics, Broad Institute of MIT and Harvard, Cambridge, MA 02142, USA; Stanley Center for Psychiatric Research, Broad Institute of MIT and Harvard, Cambridge, MA 02142, USA; Analytic and Translational Genetics Unit, Massachusetts General Hospital, Boston, MA 02114, USA; Center for Genomic Medicine, Massachusetts General Hospital, Boston, MA 02114, USA; Finnish Institute for Molecular Medicine (FIMM), University of Helsinki, Helsinki 00014, Finland

## Abstract

**Motivation:**

Biobank scale genetic associations results over thousands of traits can be difficult to visualize and navigate.

**Results:**

We have created LAVAA, a visualization web-application to generate genetic volcano plots for simultaneously considering the *P*-value, effect size, case counts, trait class and fine-mapping posterior probability at a single-nucleotide polymorphism (SNP) across a range of traits from a large set of genome-wide association study. We find that user interaction with association results in LAVAA can enrich and enhance the biological interpretation of individual loci.

**Availability and implementation:**

LAVAA is available as a stand-alone web service (https://geneviz.aalto.fi/LAVAA/) and will be available in future releases of the finngen.fi website starting with release 10 in late 2023.

## 1 Introduction

The genome-wide association study (GWAS) has been a very successful technique for elucidating the role of inherited variation in human biology and pathophysiology. The importance of a statistical association between a genetic variant and a trait of interest is conveyed by the strength of the association, reflected in the *P*-value, and the magnitude of the association, reflected in the effect size. The Manhattan plot and the regional association plot are two popular methods to explore the distribution of the *P*-values across the genome for one trait at a time (Boughton *et al.*, 2021). However, even when interpreting the results of a single GWAS at a single locus it is essential to consider that result in the context of all other genetic studies. The so-called ‘phenome-wide association scan’ or PheWAS, can be presented in a manner analogous to the Manhattan plot, showing the distribution of *P*-values across a set of traits, but now at a single variant at a time (Denny *et al.*, 2010).

As cohort sizes grow, however, it is becoming increasingly important to consider effect size in addition to the *P*-value. Indeed, in the limit we might expect that eventually all genes in relevant tissues will show statistical significance with all traits (Boyle *et al.*, 2017). Thus it is likely the case that the most important biological impact of a locus is revealed not in the trait with the strongest *P*-value but the one with the largest effect.

**Fig. 1.**
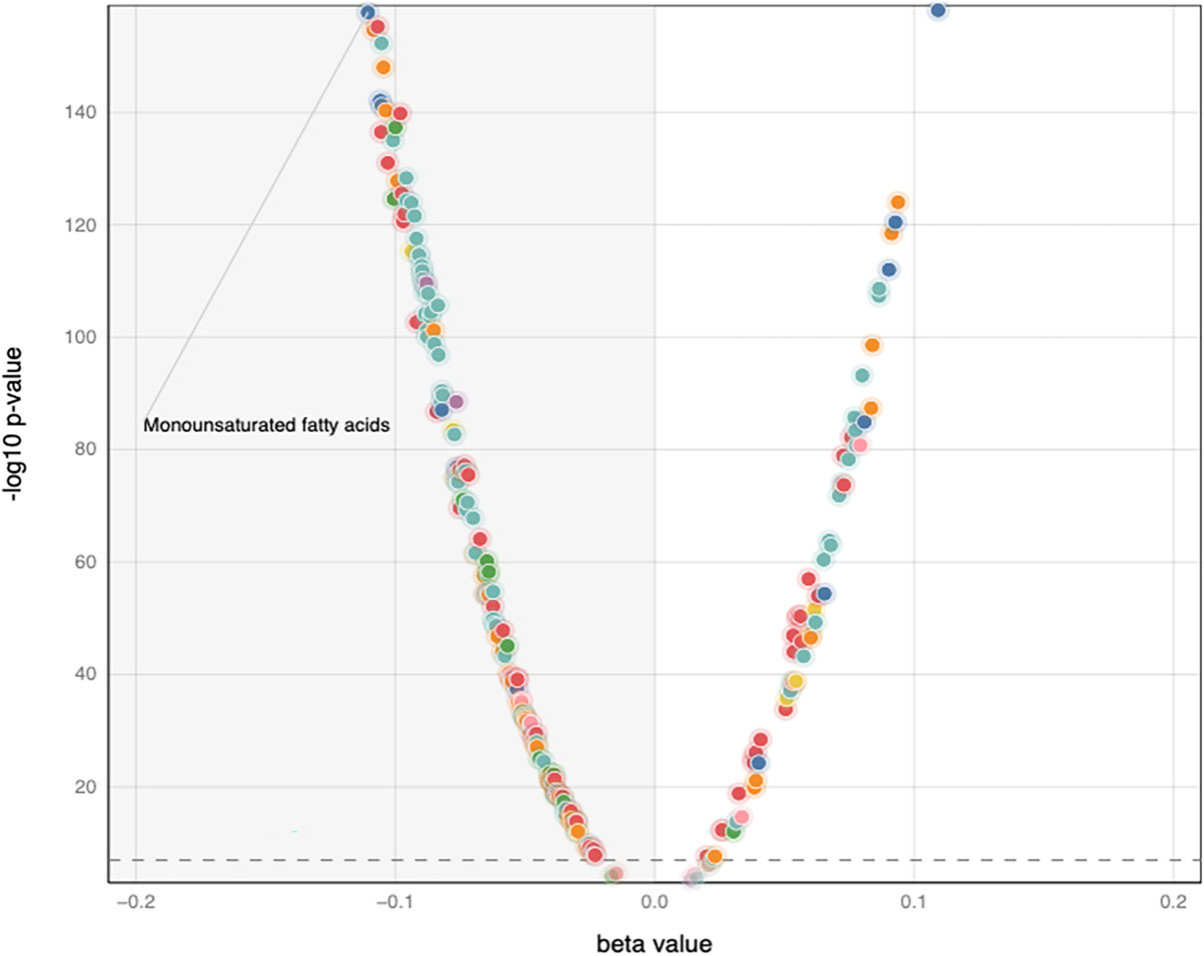
Sample LAVAA plot for a continuous trait on imported data. This LAVAA plot was created using data imported from OpenGWAS (https://gwas.mrcieu.ac.uk/) (Hemani *et al.*, 2018) representing metabolomics traits at the *GCKR* locus (rs1260326) from UK Biobank (Richardson *et al.*, 2022; Sudlow *et al.*, 2015). Each dot represents the –log10(*P*-value) and beta value for one metabolite trait. The colors represent the classes of measured traits

**Fig. 2.**
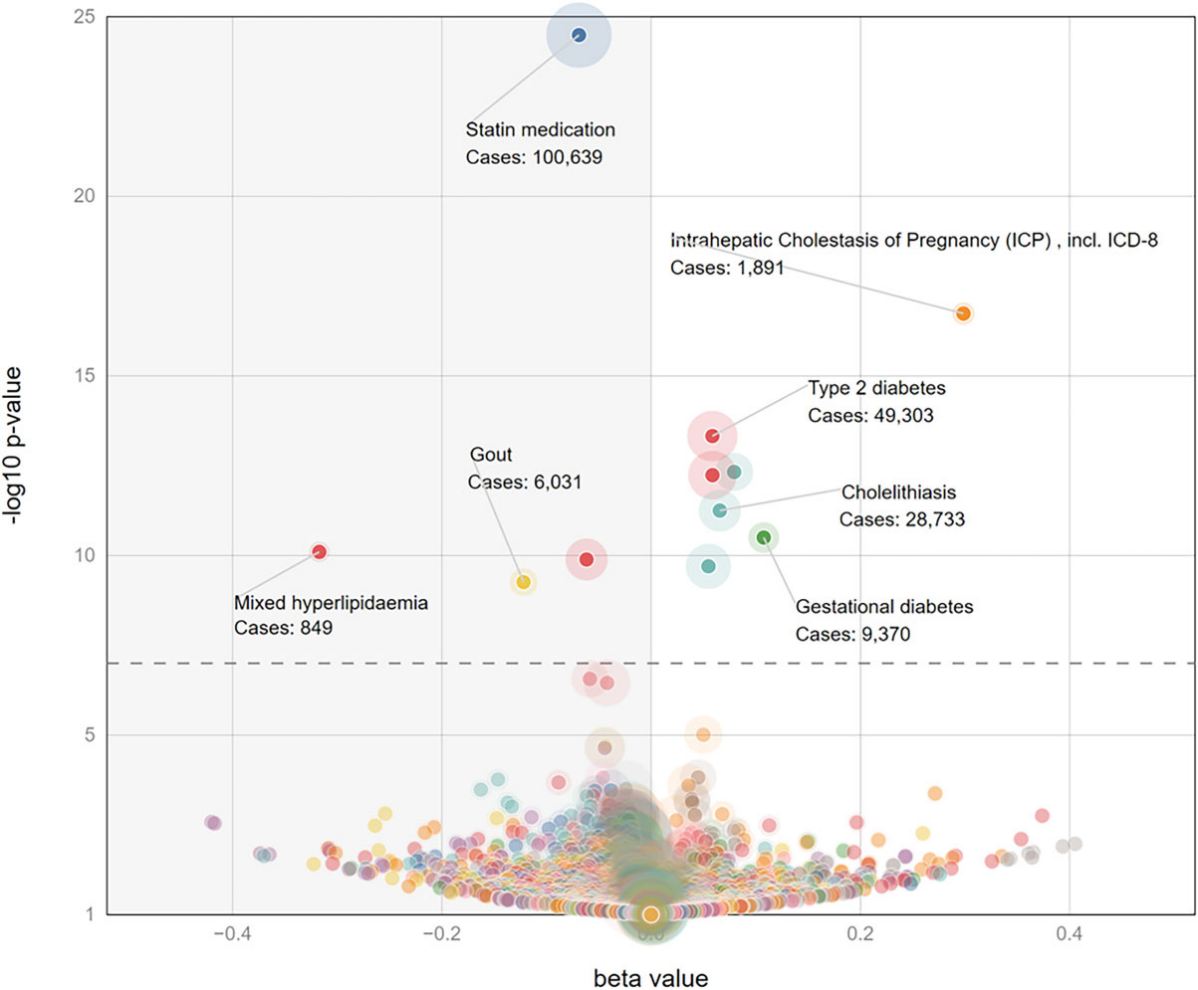
Sample LAVAA plot for a set of dichotomous traits. Since effective sample size varies drastically from trait to trait the –log10(*P*-value) and effect size are no longer coupled. This figure represents disease traits from release 7 of FinnGen at the same SNP referenced in Fig. 1. Colors represent groups of related diseases

For continuous traits (e.g. biomarker levels) at a single-nucleotide polymorphism (SNP) the trait with the strongest *P*-value will typically be the trait with the largest effect size (given a fixed sample size, cohort, and standard error across traits) (Fig. 1) (Sham and Purcell, 2014). However, this will not be the case for dichotomous traits (e.g. ‘disease’ vs ‘not-disease’), where the number of ‘cases’ can vary widely across the traits, even if the total number of subjects is constant (Fig. 2).

The genomic context of the association of a single SNP in a GWAS is also important. Because of linkage disequilibrium, the *P*-value for a trait at a particular SNP can reach genome-wide significance (*P* < 5 × 10^−8^) but may be entirely explained by a much stronger signal 10 s or 100 s of kilobases away. Statistical fine-mapping techniques (Wang *et al.*, 2020) can pinpoint likely causal variants among correlated variants, but this information is typically absent from visualizations or analyses at a specific SNP.

FinnGen is one of the largest nationwide biobank studies, now encompassing 309 154 subjects with genetic results across 3095 dichotomous traits (as of release 7) (https://r7.finngen.fi/) (Locke *et al.*, 2019). To assist researchers with extracting valuable genetic, biological and medical insights from this wealth of data we have designed and implemented LAVAA, a lightweight association viewer across ailments.

## 2 Methods

LAVAA was developed using the D3 JavaScript library. D3 provides a flexible framework and an active online community to accelerate development of new applications. We also included the following libraries: d3-tip, d3-legend and d3-format. These libraries further streamline development by adding components created specifically for use by D3-based applications. We also included the simplebar library to support an intuitive user interface design.

The LAVAA user interface is designed to support two aspects of the research process: analysis and display of results. Following ‘Shneiderman’s Mantra’ (Schneiderman, 1996) LAVAA first provides an overview of the data with a volcano plot with options to then zoom and retrieve specific details on demand. The initial plot provides the following four dimensions: (i) the magnitude of the association (the beta or the natural log of the odds ratio) on the *x*-axis; (ii) the strength of the association (the –log10 of the *P*-value from the logistic or linear regression) on the *y*-axis; (iii) the case count displayed as the diameter of the halo around each dot; and (iv) the category of each trait conveyed by the color of the halo around each dot. In this way, every association for a particular variant can be viewed and explored (Fig. 2).

LAVAA provides multiple mechanisms for the user to further explore the data. If fine-mapping results are available, traits which have been fine-mapped to the current variant can be highlighted with a dark circle around the central dot. Mousing over any dot displays the association statistics and provides an option to label the dot with those details. A user can zoom in to just the genome-wide significant results (–log10(*P*) > 7.3) or can sweep and select an arbitrary region of the display. The latter action generates a small table of the association results for all selected variants.

**Fig. 3.**
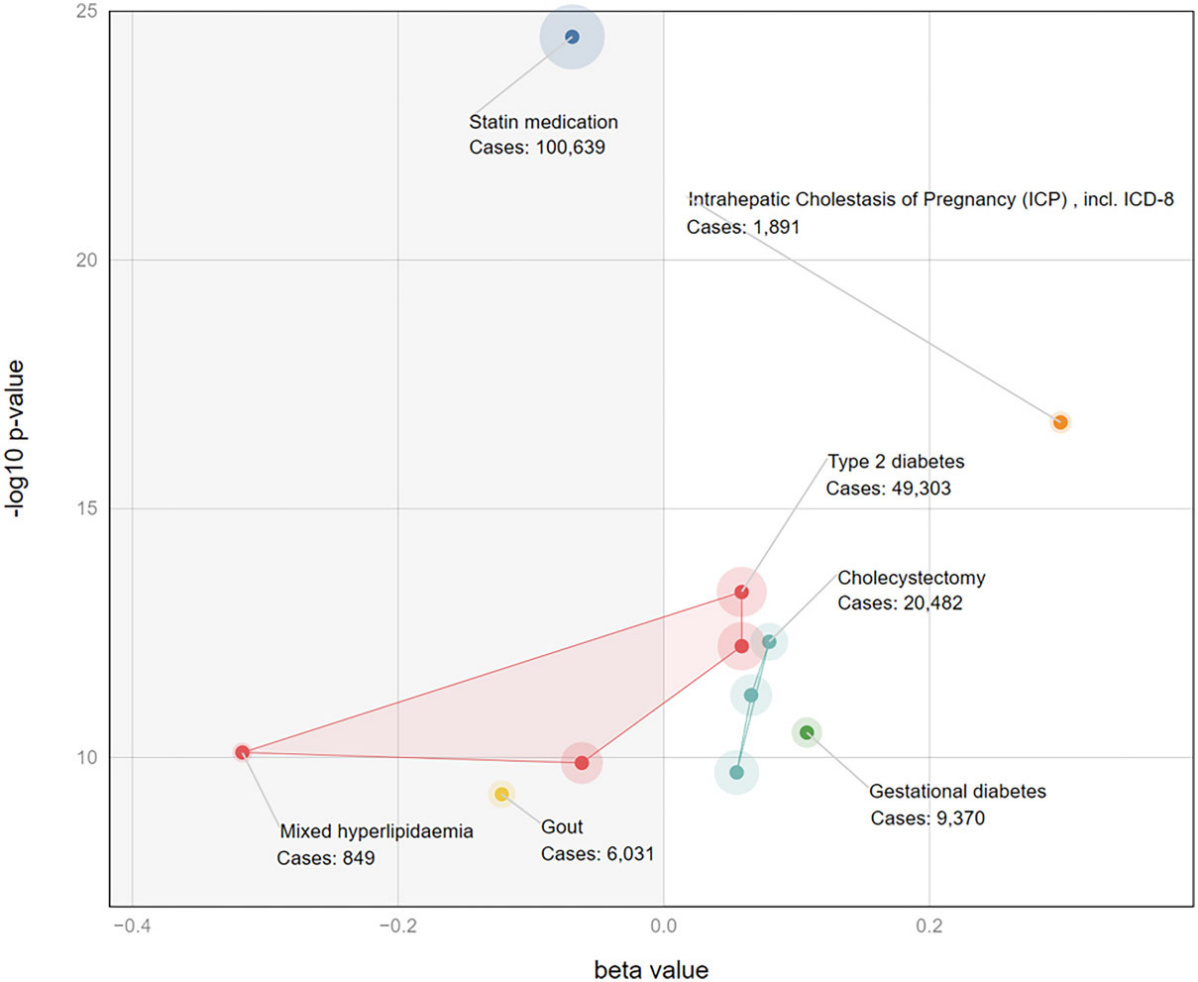
Example LAVAA plot demonstrating the use of convex hulls to group related traits. This is showing only the significant associations from Figure 2. The convex hull for ‘endocrine, nutritional and metabolic diseases’ crosses the beta = 0 line because the allele at rs1260326 which increases risk for type 2 diabetes also decreases risk for mixed hyperlipidemia

Because the thousands of traits in FinnGen have been mapped to a smaller number of specific categories we implemented a convex hull function which draws the user’s eye toward sets of related phenotypes (Fig. 3). A convex hull is defined as the smallest polygon which contains a specific set of points. By changing the focus to categories rather than individual phenotypes the convex hull provides the user with a more global view and reduces the cognitive load (Xu and Chun, 2007).

### 2.1 Interactive plots of user-provided data

Users can generate their own LAVAA plots by downloading the TSV summary of association results from any variant page in the finngen.fi website (e.g. https://r5.finngen.fi/variant/2-27508073-T-C). This TSV can be directly uploaded to the LAVAA tool here: https://geneviz.aalto.fi/LAVAA/. The LAVAA visualization tool is integrated into the finngen.fi website as of release 10, due to be available to the general public by the end of 2023.

Currently, all users can use the standalone LAVAA tool and can also download or fork the LAVAA project on GitHub, here: https://github.com/FINNGEN/volcano_plot.

## 3 Results

We demonstrate the utility of LAVAA plot with an example from the FinnGen. FinnGen employs a rich variety of case-control definitions, refining or expanding the number of subjects included. For example, the broadest definition of ‘Endocrine, nutritional and metabolic diseases’ encompasses over 118 000 cases out of 309 154 subjects. This broad category shows no significant association at the well-known *GCKR* non-synonymous variant, rs1260326 (*P* = 0.074, beta = 0.01 in FinnGen r7). However, as the LAVAA plot reveals, more specific definitions of this disease category can have much stronger associations (Fig. 2). The largest effect size for a genome-wide significant association at rs1260326 is for ‘mixed hyperlipidemia’ with only 849 cases but with an effect size of −0.32 and a *P*-value of 8 × 10^−11^. Similarly, the LAVAA plot illustrates the bifurcation of lipid and diabetic traits with the diabetes risk allele being associated with lower lipid levels and reduced use of statins (Figs 2 and 3).

## 4 Conclusion

LAVAA is a novel web-based visualization tool for assorted traits at a particular locus. By simultaneously representing multiple features per association, LAVAA permits researchers to more deeply explore biobank-scale genetic results such as from FinnGen.

## Data Availability

The data shown in Figures 1, 2, and 3 of this article are available for download from the stand-alone LAVAA server at https://geneviz.aalto.fi/LAVAA/.
